# Natural Molecules From Chinese Herbs Protecting Against Parkinson’s Disease via Anti-oxidative Stress

**DOI:** 10.3389/fnagi.2018.00246

**Published:** 2018-08-28

**Authors:** Yaqi Ding, Chenqi Xin, Cheng-Wu Zhang, Kah-Leong Lim, Hang Zhang, ZhenQian Fu, Lin Li, Wei Huang

**Affiliations:** ^1^Key Laboratory of Flexible Electronics – Institute of Advanced Materials, Jiangsu National Synergistic Innovation Center for Advanced Materials, Nanjing Tech University, Nanjing, China; ^2^Neurodegeneration Research Laboratory, National Neuroscience Institute, Singapore, Singapore; ^3^Department of Physiology, Yong Loo Lin School of Medicine, National University of Singapore, Singapore, Singapore

**Keywords:** Parkinson’s disease, dopaminergic neurons, oxidative stress, natural molecules, Chinese herb

## Abstract

Parkinson’s disease (PD) is the second most common neurodegenerative disease after Alzheimer’s disease, affecting about 7–10 million patients worldwide. The major pathological features of PD include loss of dopaminergic (DA) neurons in the substantia nigra pars compacta (SNpc) of the midbrain and the presence of α-synuclein-enriched Lewy bodies. Although the mechanism underlying PD pathogenesis remains to be elucidated, oxidative stress induced by the overproduction of reactive oxygen species (ROS) is widely accepted to be a key pathogenic factors. ROS cause oxidative damage to proteins, lipids, and DNA, which subsequently lead to neurodegeneration. Great efforts have been made to slow or stop the progress of PD. Unfortunately there is no effective cure for PD till now. Compounds with good antioxidant activity represent the promising candidates for therapeutics of PD. Some natural molecules from Chinese herbs are found to have good antioxidant activity. Both *in vitro* and *in vivo* studies demonstrate that these natural molecules could mitigate the oxidative stress and rescue the neuronal cell death in PD models. In present review, we summarized the reported natural molecules that displayed protective effects in PD. We also addressed the possible signal pathway through which natural molecules achieved their antioxidative effects and mitigate PD phenotypes. Hopefully it will pave the way to better recognize and utilize Chinese herbs for the treatment of PD.

## Introduction

Parkinson’s disease (PD) is a devastating neurodegenerative disorder characterized by progressive loss of DA neurons in the SNpc of the midbrain, affecting 1–3% of the elderly population over 60 years (De Lau and Breteler, 2006). Currently, PD remains incurable and exerts heavy socio-economic burden to the society (Whetten-Goldstein et al., 1997; Lindgren et al., 2005; Winter et al., 2010). Although the precise molecular events underlying the pathogenesis of PD remain to be elucidated, the etiology of PD is found to involve environmental factors as well as genetic predisposition (Thomas and Beal, 2007). Regardless of exogenous or endogenous factors, oxidative stress is thought to play a pivotal role in the pathogenesis of PD (Jenner, 2003; Przedborski, 2017).

Oxidative stress is caused by imbalance of pro-oxidants and anti-oxidants in the cells (Barnham et al., 2004). Major reactive oxygen species (ROS) are produced in the process of adenosine triphosphate (ATP) synthesis which occurs in the mitochondria (Shadel and Horvath, 2015). DA neurons need relatively high amount of ATP to synthesize and release dopamine (Mamelak, 2018). Hence, more ROS are produced in DA neurons compared to other types of neurons. Additionally, mitochondria are believed to contribute to the generation of ROS as a result of the accumulation of mitochondrial DNA (mtDNA) mutations during the aging process (Gredilla et al., 2012). The accumulation of mtDNA mutations could decrease the capability of the electron transport chain (ETC), triggering decreased ATP production and increased ROS production (Cha et al., 2015). Rotenone, a selective inhibitor of complex I of mitochondrial respiratory chain, was been proved to cause mitochondrial dysfunction as well as ROS accumulation. Moreover, reduced level or activity of antioxidants such as superoxide dismutase (SOD), catalase (CAT), glutathione (GSH), and glutathione peroxidase (Gpx) is another contributor to the build-up of oxidative stress (Koppula et al., 2012). Mutation of mitochondria homeostasis-related genes, including parkin, DJ-1, PTEN-induced putative kinase 1 (PINK1), peroxisome proliferator-activated receptor gamma coactivator-1α (PGC-1α), and leucine-rich repeat kinase 2 (LRRK2) has also been reported to lead to familial PD (Finck and Kelly, 2006; Handschin and Spiegelman, 2006; Aquilano et al., 2008; Schapira, 2008). Taken together, compelling evidence implicates the involvement of ROS-related stress in the pathogenesis of PD.

Currently there is no effective treatment for PD. Although L-DOPA, as the substitute of dopamine, has been widely used in clinic, the poisonous and side effect over time limits its application. Chinese herbs, which have been used for thousands of years to treat various diseases in China, represent an alternative strategy given their higher efficacy and relatively modest side effects. In PD treatment, the reported effects of Chinese herbs including antioxidant, anti-inflammatory, free radicals-scavenging, anti-apoptosis, and chelating harmful metals (Fu et al., 2015). In this review, we will discuss the role of oxidative stress in PD pathogenesis, summarize the anti-ROS effects of natural molecules from Chinese herbs and its possible mechanisms, with the view to position Chinese herbs as an alternative or complementary approach in treating PD patients.

## Oxidative Stress and PD

**FIGURE 1:**
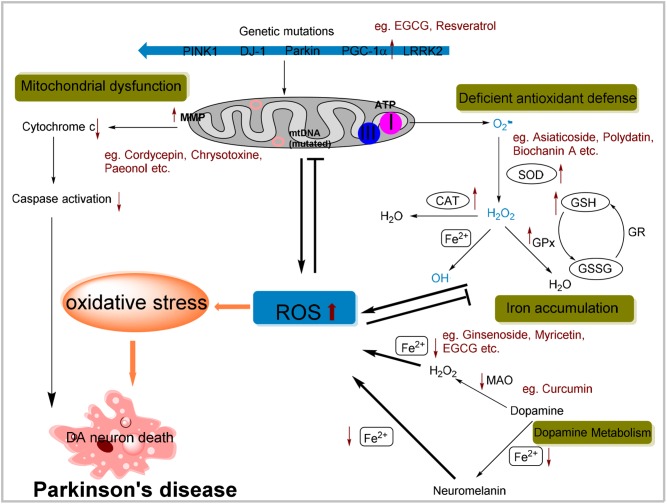
Possible cellular mechanisms attributed to oxidative stress in Parkinson’s disease (PD) and possible effects of natural antioxidants on specific pathway. Mitochondrial dysfunction by complex I inhibition and mtDNA mutation resulting in the ROS overproduction or mutation of genes involved in mitochondrial homeostasis can lead to an increased oxidative stress. ROS in turn results in the collapse of MMP and then initiates apoptosis signaling pathways contributing to DA neurons death. Also, the deficiency of antioxidant defense system may contribute to increased level of ROS. Additionally, dopamine metabolism can generate some active molecules contributing to ROS generation. Iron aggregation also enhances oxidative stress through the Fenton and Haber-Weiss reactions. In summary, all these cellular mechanisms due to the oxidative stress are implicated in the selective degeneration of dopaminergic neurons. Moreover, anti-oxidative effects of a portion of natural compounds mentioned above have been listed within specific pathway.

Oxygen is the prerequisite for nearly all forms of living organisms, but it is the source of oxidative stress also (Uttara et al., 2009). Oxidative stress results from excessive ROS, which is the consequence of imbalance between pro-oxidant and anti-oxidant homeostasis. ROS mainly comprise hydrogen peroxide (H_2_O_2_), superoxide anions (O_2_^−^) and the highly reactive hydroxyl radicals (OH^•^). Normally, the generation and elimination of ROS is well coordinated to maintain the redox status. Once the balance broken, oxidative stress will be induced, and subsequently diseases such as PD might occur. The concept of ROS involving the PD pathogenesis has been supported by multiple evidences (**Figure 1**).

Aging, iron accumulations, mitochondrial dysfunction, dopamine metabolism, which are all the established PD causative factors, meanwhile render oxidative stress. For example, incidence of PD is increased with aging, especially in elders. This is in accordance with that aged neuronal cells show hypersensitive toward oxidative stress (Floyd and Carney, 1992). The surveillance of antioxidants was also found compromised with aging (Lepoivre et al., 1994; Schulz et al., 2000). Active iron metals involved in generation of ROS through Fenton reaction and iron accumulation was attributed to PD pathogenesis (Takahashi et al., 2001; Maynard et al., 2002). Metabolism of dopamine produced many reactive molecules hence DA neurons were particularly susceptible to oxidative stress (Segura-Aguilar et al., 2014). Dopamine was synthesized in the cytoplasm and immediately sequestered into monoaminergic vesicles (Herrera et al., 2017). If not secreted, dopamine could be auto-oxidized or deaminated by monoamine oxidase (MAO), during which several cytotoxic molecules, including dopamine–quinone species (SQ^•^), OH^•^, and H_2_O_2_ are generated (Graham, 1978; Maker et al., 1981). Notably, 1-methyl-4-phenyl-1,2,3,6-tetrahydropyridine (MPTP) is commonly used to induce oxidative stress-related Parkinsonism in animal models. The active metabolite of MPTP, MPP^+^, was selectively taken up into dopaminergic terminals by the plasma-membrane dopamine transporter (DAT) (Choi et al., 2015). MPP^+^ would block complex I activity of the mitochondrial respiratory chain and result in the oxidative stress (Del Zompo et al., 1992; Peter et al., 1994; Lotharius and O’Malley, 2000).

Mitochondrial dysfunction, resulting from various malgenic factors, has been implicated in ROS generation and oxidative stress in PD. mtDNA mutations contributed to mitochondrial dysfunction due to impaired capability of the ETC, triggering ROS production (Gredilla et al., 2012; Yan et al., 2013; Cha et al., 2015). ROS in turn resulted in the collapse of the mitochondrial membrane potential (MMP) and disruption of the mitochondrial respiratory chain complex I, which ultimately leaded to increased cytosolic concentrations of Ca^2+^ and mitochondrial cytochrome c that initiated apoptosis signaling pathways (Du et al., 2010). Mutation of genes involved in mitochondrial homeostasis was known to induce familial PD (Schapira, 2007). Parkin, an E3 ubiquitin ligase, was mitochondria key regulator of mitophagy (Narendra et al., 2008). Not surprisingly, Drosophila deficient in parkin, exhibited mitochondrial dysfunction and higher vulnerability to oxidative stress (Whitworth et al., 2005; Ng et al., 2012). Parkin knockout mice showed decreased amounts of several proteins involved in mitochondrial function and enhanced oxidative stress (Palacino et al., 2004). PINK1, together with Parkin, were tightly coordinated to the controlling of mitochondrial dynamics (Scarffe et al., 2014). PINK1 accumulates on the outer membrane of damaged mitochondria and recruits Parkin to the dysfunctional mitochondrion (Pickrell and Youle, 2015). It was showed that the lack of PINK1 resulted in the mitochondrial defects and loss of SNpc DA neurons, and these phenomena could be ameliorated by the enhanced expression of Parkin (Yang et al., 2006; Gautier et al., 2008). Many lines of evidence suggested that DJ-1, another gene reported to cause a familial early onset PD, functions as an antioxidant protein. DJ-1 bonded to subunits of mitochondrial complex I and regulates its activity (Hayashi et al., 2009). DJ-1 mutation promoted its accumulation in mitochondria and was implicated as a cellular monitor of oxidative stress (Mitsumoto and Nakagawa, 2001; Bonifati et al., 2002). DJ-1 knockout mice displayed compromised mitochondrial function and then nigrostriatal DA neuron loss (Goldberg et al., 2005; Giaime et al., 2012). Furthermore, dysfunction of PGC-1α, the key transcriptional modulator of mitochondrial biogenesis and oxidative metabolism, was also implicated in PD (Arany et al., 2005; Lin et al., 2005). PGC-1α regulated the mitochondrial function and provides homeostatic control of cellular ATP (Rohas et al., 2007). It was revealed that inhibited expression of PGC-1α resulting from methylation of its gene contributed to the mitochondrial defects in substantia nigra of PD patients (Su et al., 2015). Dominant mutations in LRRK2 are currently recognized to be the most prevalent cause of late-onset familial PD (Kumari and Tan, 2009). Actually LRRK2 patient-derived cells exhibited altered mitochondrial dynamics that was accompanied by reduction in MMP and intracellular ATP levels (Mortiboys et al., 2010). Moreover, Drosophila with LRRK2 G2019S mutant induced marked mitochondrial pathological alternation both in flight muscles and DA neurons (Ng et al., 2012).

## Natural Anti-Oxidant Molecules and Their Applications in PD Models

Vast territory of China has brought about abundant natural resources. One precious natural resource is Chinese herb, which has been used throughout history to improve quality of human life. In recent years, traditional Chinese herbs attract more interests due to its impressive curative effect for a variety of diseases, coupled with lower toxicity and side effects (Fu et al., 2015). As mentioned above, oxidative stress damage is one of the most important characteristics of PD. Natural antioxidants, which are enriched in Chinese herbs, provide neuroprotective effects in PD through a variety of biological pathways (Obrenovich et al., 2010; Soobrattee et al., 2010). In this review, polyphenols, flavone (baicalein), flavonols (quercetin, kaempferol, morin, and myricetin), dihydroflavones (hesperetin and naringenin), isoflavone (biochanin A), and flavane (epigallocatechin gallate), and other non-flavonoids phenolic compounds (resveratrol, curcumin, and paeonol) from Chinese herbs against PD will be discussed. Moreover, the well-known glycoside derivatives [mangiferin, salidroside, asiaticoside, polydatin, gypenoside (GP), and ginsenoside], and other compounds (nerolidol, chrysotoxine, DL-3-n-butylphthalide, cordycepin, and ursolic acid) from natural resources with good antioxidant properties will also be addressed to discuss their possible mechanisms of against PD (**Table 1**).

**Table 1:** Natural anti-oxidant molecules from Chinese herbs against PD.

Substances	Studied models *in vivo* or *in vitro*	Possible mechanisms	Reference
**1. Modulate mitochondrial function**			
Epigallocatechin gallate (EGCG)	MPP^+^-treated PC12 cells	Up-regulates PGC-1α and improves mitochondrial function.	Ye et al., 2012
	Drosophila with mutant LRRK2 and null parkin	Attenuates mitochondrial-associated pathway in LRRK2 and parkin-related pathogenesis.	Ng et al., 2012
Resveratrol	Primary fibroblast from PD patients with PARK2 mutation	Raises the expression of PGC-1α’s target genes (TFAM, cytochrome c and COX I).	Ferretta et al., 2014
Cordycepin	6-OHDA-induced PC12 cells	Maintains mitochondrial membrane potential (MMP) and reduce activation of caspase-3.	Olatunji et al., 2016
Baicalein	6-OHDA-induced SH-SY5Y cells	Attenuates mitochondrial dysfunction, oxidative injury, JNK and caspase activation.	Lee et al., 2005
Curcumin	MPP^+^-induced PC12 cells	Mediates the toxicity of PC12 through Bcl-2-mitochondria-ROS-iNOS pathway.	Chen et al., 2006
Chrysotoxine	6-OHDA-induced SH-SY5Y cells	Attenuates the decrease of MMP, release of cytochrome c, imbalance of Bax/Bcl-2 ratio and activation of caspase-3.	Song et al., 2010
DL-3-n-butylphthalide (NBP)	MPP^+^-induced PC12 cells	Retains mitochondrial function and suppresses ROS generation.	Huang et al., 2010
Mangiferin	Rotenone-induced SK-N-SH cells	Rectifies oxidative imbalance and protects mitochondrial function.	Kavitha et al., 2014
Morin	Excitotoxic neuron with NMDA receptor over-activation	Reduces ROS by restoring the MMP.	Campos-Esparza et al., 2009
Paeonol	MPP^+^-induced mice	Restores MMP and reduces cytochrome c release and caspase-3 activity.	Lu et al., 2015
Ursolic acid	Parkin-mutant fibroblasts	Rescues mitochondrial function by the activation of the glucocorticoid receptor with increased phosphorylation of Akt.	Mortiboys et al., 2013
Salidroside	MPP^+^-induced PC12 cells	Regulates the ratio of Bcl-2/Bax, decrease cytochrome c and Smac release, and inhibit caspase activation.	Wang S. et al., 2015
**2. Activate intracellular antioxidants**			
Asiaticoside	MPTP-induced rats	Attenuates the reduction of GSH level in the substantia nigra.	Xu et al., 2012
Polydatin	Rotenone-induced rats	Increases the level of GSH and manganese superoxide dismutases (MnSOD) in the striatum.	Chen Y. et al., 2015
Biochanin A	Lipopolysaccharide (LPS)-induced rat	Increases SOD and Gpx activities in the midbrain tissue.	Wang J. et al., 2015
	L-glutamate-treated PC12 cells	Increases total GSH activities.	Tan et al., 2012
Gypenosides (GP)	MPTP-induced mice	Attenuates the decrease of GSH content and SOD activities in the substantia nigra.	Wang et al., 2010
Mangiferin	MPP^+^-induced N2A cells	Restores the GSH content and down-regulates both SOD1 and CAT mRNA expression.	Amazzal et al., 2007
Nerolidol	Rotenone-induced rats	Increases the level of SOD, CAT, and GSH in midbrain cells.	Javed et al., 2016
Quercetin	6-OHDA-induced rats	Restores the level of GSH in the striatum.	Haleagrahara et al., 2011
	H_2_O_2_-induced PC12 cells	Reduces CAT, SOD and Gpx level.	Chen L. et al., 2015
Kaempferol	MPTP-induced mice	Increases SOD and Gpx activities in the substantia nigra.	Żuk et al., 2011
Cordycepin	6-OHDA-induced PC12 cells	Increases SOD and Gpx activities.	Olatunji et al., 2016
Resveratrol	6-OHDA-induced rats	Up-regulates GPx, GR, CAT, and SOD activities.	Khan et al., 2010
Paeonol	MPTP-induced mice	Enhances the levels of SOD, CAT, and GSH.	Shi et al., 2016
Gastrodin	MPP^+^-induced oxidative PD model	Increases antioxidant enzyme HO-1 expression.	Jiang et al., 2014
Hesperetin	6-OHDA-lesioned rats	Enhances striatal CAT and GSH content.	Kiasalari et al., 2016
**3. Mediate metabolism of dopamine**			
Curcumin	MPTP-induced rats	Inhibits MOA-B activity.	Rajeswari and Sabesan, 2008
**4. Decrease iron metal levels**			
Curcumin	6-OHDA-induced rats	Chelates iron metals in the substantia nigra.	Du et al., 2012
Ginsenoside	6-OHDA-induced MES23.5 cells	Inhibits up-regulation of an iron importer protein DMT1 with iron IRE.	Xu et al., 2010
Myricetin	6-OHDA-induced rats	Prevents the increase of iron-staining cells in the substantia nigra.	Ma et al., 2007
EGCG	Iron-induced SH-SY5Y cells	Alleviates the iron accumulation through affecting IRE.	Reznichenko et al., 2006
Naringenin	Iron-induced rats	Chelates iron metals in the cerebral cortex.	Chtourou et al., 2014

### Natural Molecules Modulating Mitochondrial Function

Mitochondrial dysfunction-induced oxidative stress is widely accepted to be the key driver of PD. Epigallocatechin gallate (EGCG) is an important component of green tea and it has lots of biological effects, such as antioxidation, scavenging of free radicals and anti-apoptosis. In MPP^+^-treated PC12 cells, EGCG caused up-regulation of PGC-1α, resulting in improved mitochondrial function and DA neuronal survival (Ye et al., 2012). In addition, EGCG was reported to act as a suppressor of mitochondrial dysfunction in both mutant LRRK2 and parkin-null flies through activation of the AMP-activated protein kinase (AMPK) signaling pathway (Ng et al., 2012). Resveratrol, a polyphenolic compound enriched in grapes, was shown to improve mitochondrial activity via affecting energy metabolic sensors through mediation of autophagy signals and activation of NAD-dependent deacetylase sirtuin-1 (SIRT1) and PGC-1α (Lagouge et al., 2006; Jeong et al., 2012). Resveratrol could also enhanced the mRNA level of a number of PGC-1α target genes such as mitochondrial transcription factor A (TFAM), cyclooxygenase-1 (COX I), resulting in mitochondrial biogenesis (Ferretta et al., 2014). Cordycepin, a nucleoside isolated from *Cordyceps* species displayed antioxidant property. Pretreatment of cordycepin helped to maintain MMP and reduced activation of caspase-3 in 6-OHDA induced PD model (Olatunji et al., 2016). The flavone baicalein, isolated from *Scutellaria baicalensis*, was reported to protect against 6-OHDA-induced neurotoxicity both *in vivo* and *in vitro* via confronting mitochondrial dysfunction, oxidative injury, JNK activation and caspase activation (Lee et al., 2005). Curcumin, the natural polyphenol compound derived from the curry spice turmeric, displayed neuroprotective effects on the MPTP induced PD cellular model through mediating Bcl-2-mitochondria-ROS-iNOS pathway (Chen et al., 2006). Chrysotoxine, a bioactive bibenzyl compounds isolated from medicinal *Dendrobium* species, was reported to be free radical scavengers (Zhang et al., 2007). Pretreatment with Chrysotoxine protected against 6-OHDA-induced intracellular generation of ROS and mitochondrial dysfunctions, including the decrease of MMP, increase of intracellular free Ca^2+^, release of cytochrome c, and imbalance of Bax/Bcl-2 ratio (Song et al., 2010). DL-3-n-butylphthalide (NBP), derived from l-3-n-butylphthalidec extracted from the seeds of *Apium graveolens* Linn (Chinese celery), was found to be a natural free radical scavenger (Li et al., 2009). In a cellular PD model, pretreatment with NBP mitigated the toxicity of MPP^+^ by retaining the mitochondrial function, and suppressing ROS generation (Huang et al., 2010). Two polyphenols, mangiferin and morin, specifically enriched in fruit, vegetables, plant extracts, wine, and tea, were reported to reduce the formation of ROS by restoring the MMP in excitotoxic induced cell model (Campos-Esparza et al., 2009; Kavitha et al., 2014). Paeonol, a major phenolic compound of the Chinese herb, *Cortex Moutan*, is known for its antioxidant, anti-inflammatory and antitumor properties. Paeonol has been shown to attenuate the intracellular ROS accumulation and associated mitochondrial cell death pathway including MMP disruption, cytochrome c release and caspase-3 activation in MPP^+^-induced cellular PD model (Lu et al., 2015). From high throughput screening, the natural compound ursolic acid, a pentacyclic triterpenoid, was found to rescue mitochondrial function in parkin-mutant fibroblasts via the activation of the glucocorticoid receptor that is associated with increased phosphorylation of Akt (Mortiboys et al., 2013). Salidroside (Sal), a phenylpropanoid glycoside isolated from *Rhodiola rosea L*., had potent antioxidant properties. Sal pretreatment protected DA neurons against MPP^+^-induced toxicity by reducing the production of ROS or NO, regulating the ratio of Bcl-2/Bax, decreasing cytochrome c and Smac release, and inhibiting caspase activation (Wang J. et al., 2015).

### Natural Molecules Regulating Endogenous Antioxidants and Dopamine Metabolism

Cells have endogenous defense mechanisms against oxidative stress, including enzymatic and non-enzymatic systems (Gilgun-Sherki et al., 2001). The capacity of antioxidant defenses declined with aging and in pathological state (Sohal and Weindruch, 1996; Lotharius et al., 2002). It was reported that polyphenols could modulate the activity of enzymes involved in oxidative stress (Ebrahimi and Schluesener, 2012). Xu et al. (2012) showed that asiaticoside, a triterpenoid saponin isolated from *Centella asiatica* attenuated the MPTP-induced the reduction of GSH in a rat model of Parkinsonism. Polydatin, a glycosylated derivative of resveratrol, significantly prevented the rotenone-induced decreased levels of GSH and the manganese superoxide dismutases (MnSOD) in the striatum of rodent models of PD (Chen Y. et al., 2015). Biochanin A, an *O*-methylated isoflavone found in chickpea, increased SOD and Gpx activities in lipopolysaccharide (LPS) induced rat PD model (Wang J. et al., 2015). Another research group reported that pretreatment with biochanin A could lead to the increase in the total GSH level in the L-glutamate-treated PC12 cells (Tan et al., 2012). Treatment with GPs, saponins extracted from *Gynostemma pentaphyllum*, attenuated MPTP-induced decrease of GSH and reduced SOD activity in the SNpc of the mice (Wang et al., 2010). Pretreatment with mangiferin protected N2A cells against MPP^+^-induced cytotoxicity, restored the GSH, and down-regulated both SOD1 and CAT mRNA expression (Amazzal et al., 2007). Nerolidol, a sesquiterpene alcohol, significantly increased the level of SOD, CAT, and GSH in a rotenone-induced PD experimental model (Javed et al., 2016). Quercetin, enriched in abundance in fruits and vegetables, onions, red wine and olive oil, restored level of GSH in the striatum of rats induced by 6-OHDA (Miean and Mohamed, 2001; Haleagrahara et al., 2011). In H_2_O_2_-induced PC12 cells, pretreatment with quercetin markedly reduced the antioxidant enzyme SOD and Gpx level (Chen L. et al., 2015). Kaempferol, a prototype flavonol presented in tea, broccoli, grapefruit, brussel sprouts and apple, was reported to have strong antioxidant and anti-inflammatory properties, and enhanced SOD and Gpx activity in the mouse model of PD (Żuk et al., 2011; Li and Pu, 2011). Cordycepin, resveratrol, hesperetin rendered up-regulation of the level and activity of antioxidants such as, SOD, GPx, CAT, GSH in 6-OHDA induced PD models (Khan et al., 2010; Kiasalari et al., 2016; Olatunji et al., 2016). Treatment with paeonol improved the MPTP-induced the oxidative stress, as determined by enhancing the activity levels of SOD, CAT, and GSH in the mouse PD model (Shi et al., 2016). Gastrodin, the major active component in the *Gastrodia elata*, has been demonstrated to have many pharmacological effects, such as antioxidative and neuroprotective properties. In MPP^+^-induced oxidative cellular PD model, pretreatment with gastrodin increased antioxidant enzyme heme oxygenase-1 (HO-1) expression (Jiang et al., 2014) and activation of HO-1 resulted in increased levels of antioxidant substrates such as biliverdin, bilirubin, and ferritin (Doré et al., 1999). Curcumin and its metabolite tetrahydrocurcumin (ThC) exerted neuroprotection against MPTP induced neurotoxicity via inhibiting MAO-B activity (Rajeswari and Sabesan, 2008).

### Natural Molecules Chelating Metal Iron

Iron, which accumulated in the aging brain especially the SNpc, is thought to promote PD pathogenesis (Devos et al., 2014). Iron enhances ROS generation through the Fenton and Haber-Weiss reactions (Barnham et al., 2004). Curcumin pretreatment reversed iron-induced degeneration of nigral DA neurons by its iron chelating activity (Du et al., 2012). Ginsenoside, the active component isolated from ginseng, was reported to decrease the 6-OHDA-induced iron influx by inhibiting up-regulation of an iron importer protein divalent metal transporter 1 with iron responsive element (DMT1+IRE), which was rendered via its antioxidant effect (Xu et al., 2010). Myricetin, a natural flavonoid found in fruits, vegetables, and herbs (Kang et al., 2010), was reported to suppress iron induced toxicity in the 6-OHDA induced PD model (Ma et al., 2007). EGCG served as iron chelator inhibiting the formation of transition metal catalyzed free radicals and displaying its antioxidant and neuroprotective effects (Weinreb et al., 2009; Singh et al., 2016). EGCG could also alleviate the iron accumulation in PD through affecting the iron responsive element (Reznichenko et al., 2006). Administration of naringenin (NGEN), a natural flavonoid compound, attenuated oxidative damages in the cerebral cortex of iron treatment induced PD model, due to its iron chelating activity (Chtourou et al., 2014).

## Conclusion and Perspectives

In recent years, given the limitations of current PD treatment, more attention has been given to the potential therapeutic effects of Chinese herbs (Song et al., 2012). In this review, we summarized natural antioxidants from Chinese herbs that were reported to protect against toxins-induced PD in preclinical animal models. These natural antioxidants achieve their protective effects mainly through regulating cellular oxidative homeostasis either directly or indirectly. Of them, resveratrol and EGCG, showed prominent antioxidative effect in PD models and could be a promising candidate for treating PD. Notwithstanding that, there are several challenges to overcome before natural molecules from Chinese Herbs could serve as alternative medicine for PD. How to efficiently screen and select the candidates that can be used for treatment of PD from the massive number of Chinese herbs available? What are the intracellular targets of the natural molecules? How to help the molecules pass through the blood–brain barrier (BBB) in order to directly protect the DA neurons in the SNpc? Clearly, it is promising but a lot of work needs to be done to ultimately achieve the goals of making natural molecules to be accepted and applied in PD patient treatment.

## Author Contributions

YD prepared the manuscript. CX, HZ, ZF, and WH provided help in the manuscript preparation. CW-Z, LL, and K-LL did the revision.

## Conflict of Interest Statement

The authors declare that the research was conducted in the absence of any commercial or financial relationships that could be construed as a potential conflict of interest.
